# Crystal structure, Hirshfeld surface analysis, DFT and mol­ecular docking studies of 4′-(benz­yloxy)-[1,1′-biphen­yl]-3-carb­oxy­lic acid

**DOI:** 10.1107/S2056989025001021

**Published:** 2025-02-11

**Authors:** M. Harish Kumar, M. Vinduvahini, H. T. Srinivasa, H. C. Devarajegowda, B. S. Palakshamurthy

**Affiliations:** a Department of Physics Yuvaraja's College University of Mysore,Mysore 570005 Karnataka India; b Department of Physics, Maharani’s Science College for Women (Autonomous) Mysore, Karnataka, 570005, India; c Raman Research Institute, C V Raman Avenue Sadashivanagar Bangalore Karnataka India; d Department of PG Studies and Research in Physics Albert Einstein Block UCS Tumkur University, Tumkur Karnataka 572103 India; Institute of Chemistry, Chinese Academy of Sciences

**Keywords:** crystal structure, Hirshfeld surface, DFT studies, mol­ecular docking, benz­yloxy, biphenyl carb­oxy­lic acid

## Abstract

In the title compound, C_20_H_16_O_3_, intra­molecular C— H⋯O hydrogen bonds are observed. The dihedral angles between the aromatic benzoic acid ring and the two adjacent aromatic rings are 26.09 (4) and 69.93 (8)°, while the dihedral angle between the aromatic rings connected by the C—O—C—C [torsion angle = −175.9 (2)°] link is 89.11 (3)°. In the crystal, inversion dimers linked by pairs of O—H⋯O hydrogen bonds generate *R*_2_^2^(8) ring motifs. These dimers are further linked by C—H⋯π inter­actions, forming mol­ecular sheets along (010). The mol­ecular structure was optimized by density functional theory (DFT) at the B3LYP/6–311+ G(d,p) level and the bond lengths, angles and torsion angles were compared with experimental values obtained by X-ray diffraction. The HOMO and LUMO were calculated, the energy gap between them being 4.3337 eV. Further, the inter­molecular inter­actions were qu­anti­fied using Hirshfeld surface analysis and fingerprint plots and energy frameworks were generated. The two-dimensional fingerprint plots indicate that the major contributions to the crystal packing are from H⋯H (39.7%), H⋯C (39.0%) and H⋯O (18.0%) inter­actions. The energy framework calculations reveal that the dispersion energy (*E*_dis_= 201.0 kJ mol^−1^) dominates the other energies. Mol­ecular docking studies were carried out for the title compound as a ligand and the SARS-Covid-2 (PDB ID:8BEC) protein, specifically the Omicron variant, was used as a receptor giving a binding affinity of −7.6 kcal mol^−1^.

## Abstract 2

The title mol­ecule was studied by single-crystal X-ray analysis to determine its mol­ecular structure and investigate the inter­actions present. Theoretical (obtained by DFT) and experimental parameters were compared. In addition, Hirshfeld surface analysis and mol­ecular docking studies were performed for the title compound as a ligand and the SARS-Covid-2 (PDB ID:8BEC) protein, specifically the Omicron variant.

## 1. Chemical context

The biphenyl moiety forms an important inter­mediary of compounds having profound pharmacological activities (Jain *et al.*, 2017 ▸). Biphenyl-derived drugs are found to exhibit anti-cancer, anti-diabetic, anti-inflammatory and various therapeutic activities, and represent a well-known rigid core moiety in pharmacological applications. Biphenyl carb­oxy­lic acid derivatives have been described as a new class of anti-resorptive drugs with potential therapeutic benefits for preventing and treating diseases associated with osteoclast activation such as osteoporosis, cancer-induced bone disease and Paget’s disease (Idris *et al.*, 2009 ▸; van’t Hof *et al.*, 2004 ▸) and exhibit anti-hypertensive activity (Sharma *et al.*, 2010 ▸). Biphenyl-2-carb­oxy­lic acid and biphenyl-4-carb­oxy­lic acids exhibit different levels of activity in cell toxicity tests and inhibit the tubulin polymerization process (Mukherjee *et al.*, 2016 ▸; Mahale *et al.*, 2014 ▸). Hydrazide-hydrazone-containing biphenyl compounds demonstrate potential anti-microbial activity (Deep *et al.*, 2010 ▸). Biphenyl imidazole derivatives exhibit excellent anti­fungal activity (Zhao *et al.*, 2017 ▸) while benz­yloxy triazole derivatives display moderate-to-excellent anti­bacterial activity (Kaushik *et al.*, 2018 ▸), The organic nitrate-containing benz­yloxy isonipecotanilide derivatives exhibit strong NO-mediated vasodilatory effects on pre-contracted rat aorta strips (de Candia *et al.*, 2015 ▸), and studies on bez­yloxy oxopyridin benzoate derivatives have revealed that further investigations on these compounds could lead to new compounds that may be considered as anti-malarial or cytotoxic agents (Mohebi *et al.*, 2022 ▸). As part of our studies in this area, our team is working to explore crystal structures of inter­est for biological studies.
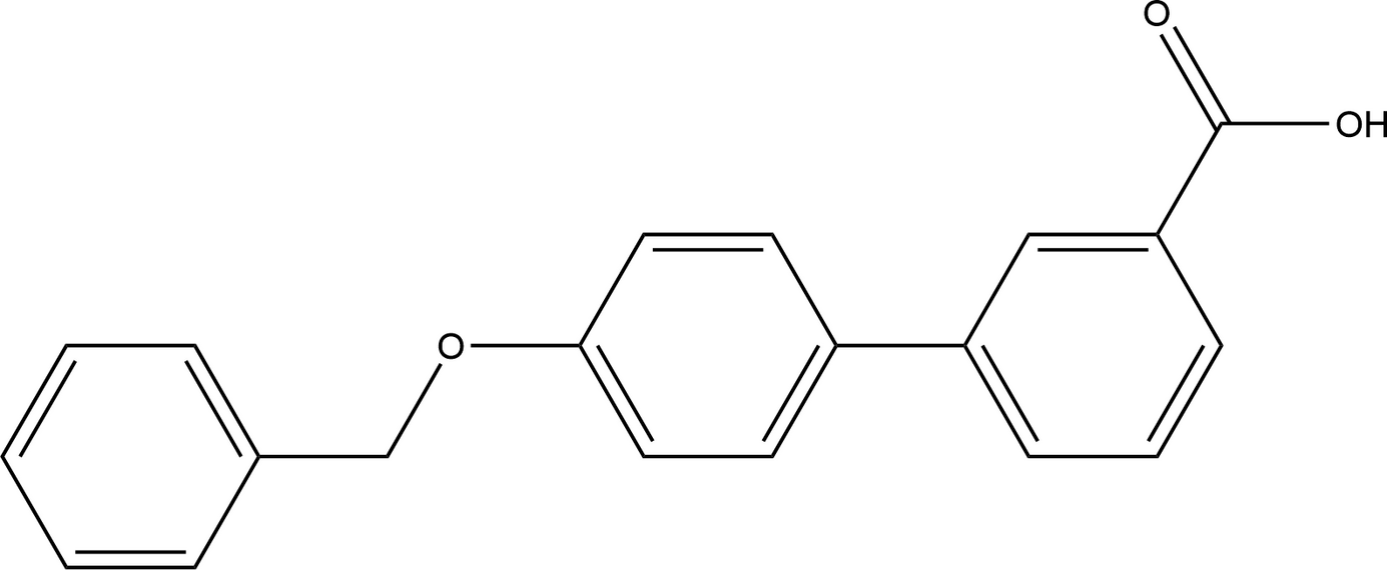


## 2. Structural commentary

**Figure 1:**
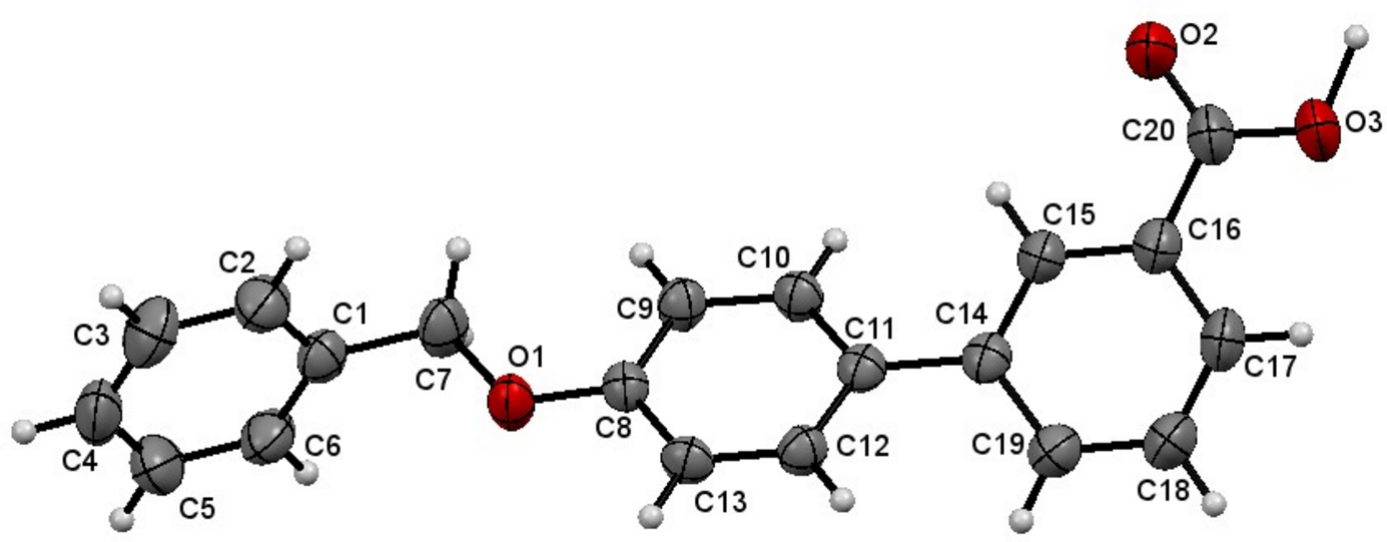
Mol­ecular structure of the title compound, showing the atom-labeling scheme. Displacement ellipsoids are drawn at the 50% probability level.

The structure of the title compound is shown in Fig. 1 ▸. The dihedral angle between the aromatic ring of the benz­yloxy group (C1–C7) and the (C8–C13) ring in the biphenyl carb­oxy­lic acid group is 89.11 (2)°, while the angle between the benz­yloxy group (C1–C7) and the (C14–C19) ring in the biphenyl carb­oxy­lic acid group is 69.93 (8)°. The dihedral angle between the adjacent rings within the biphenyl carb­oxy­lic acid group (C8–C13 and C14–C19) is 26.09 (4)°. The torsion angle within the benz­yloxy moiety (C1—C7—O1—C8) is −175.9 (2)°. Otherwise, the bond distances and angles may be regarded as normal. Intra­molecular C—H⋯O hydrogen bonds occur.

## 3. Supra­molecular features

**Figure 2:**
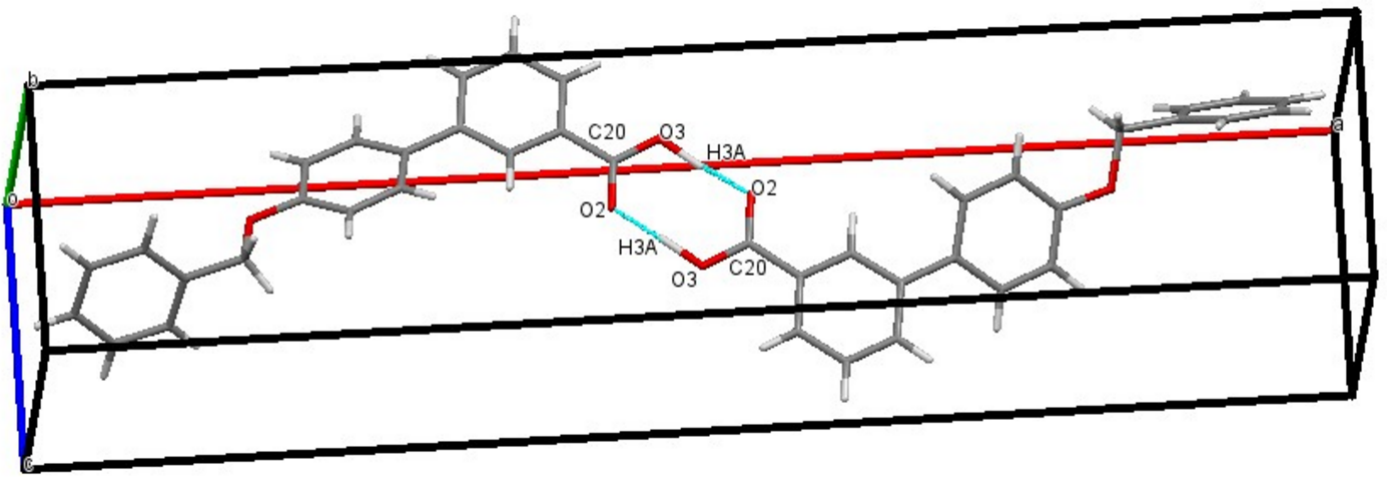
Mol­ecular packing of the title compound, showing the O—H⋯O hydrogen bonds that generate inversion dimers with 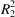
(8) ring motifs.

**Figure 3:**
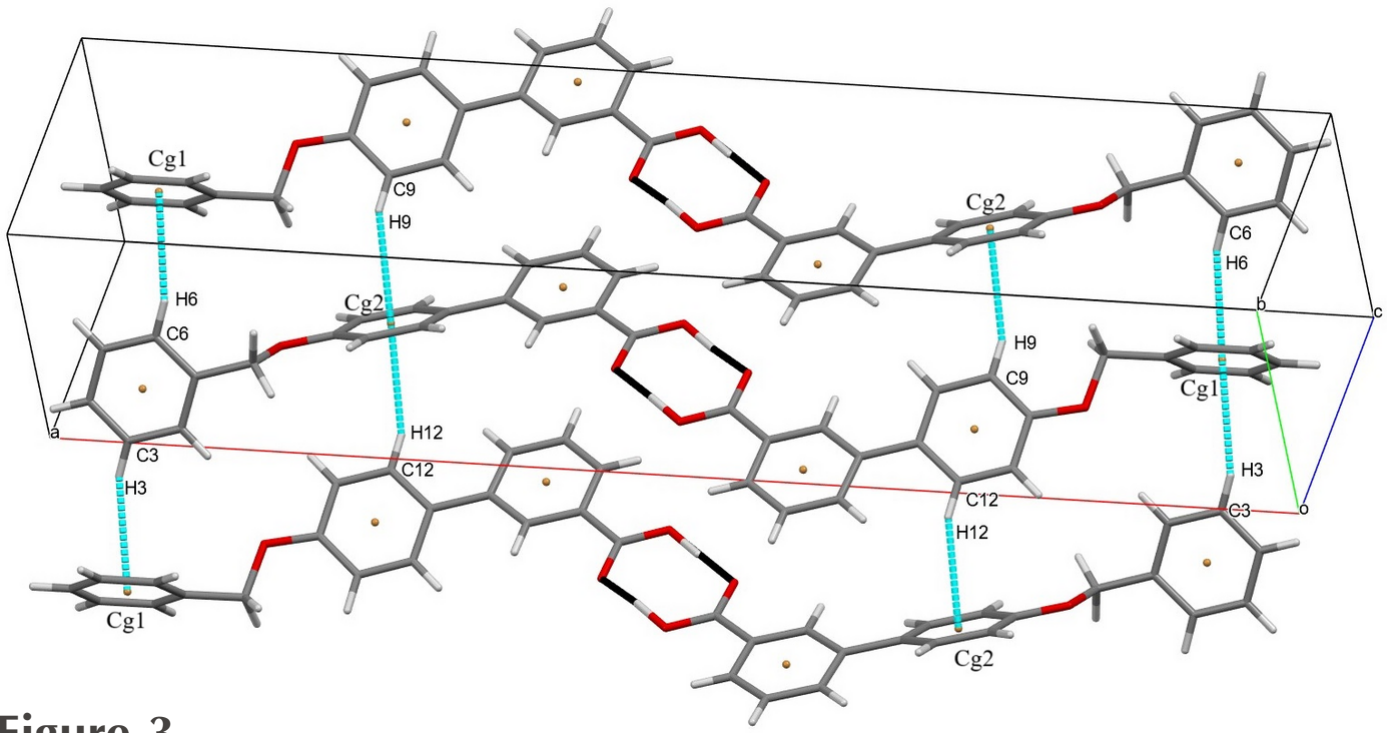
Packing of the molecules showing C—H⋯π inter­actions.

In the crystal, weak O3—H3*A*⋯O2 hydrogen bonding leads to the formation of inversion dimers, which are linked by pairs of O—H⋯O hydrogen bonds generating an 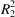
(8) ring motif (Fig. 2 ▸, Table 1 ▸). The O3—H3*A* distance of 1.20 (5) Å is quite a large as a result of tensile stress between the dimers. The tensile force between the two dimers can increase the donor–hydrogen distance, obviously weakening the hydrogen bond. In addition, the packing is consolidated by four C—H⋯π inter­actions (Table 1 ▸, Fig. 3 ▸).

## 4. Database survey

A search of the Cambridge Structural Database (CSD version 2.0.4, December 2019; Groom *et al.*, 2016 ▸) for mol­ecules containing [1,1′-biphen­yl]-3-carb­oxy­lic acid resulted in eleven matches. Of these, five compounds, CUFYEL (Guo *et al.*, 2024 ▸), HUJZIY, HUJZOE and HUJZUK (O’Malley *et al.*, 2020 ▸) and SEBMOF (Barbas *et al.*, 2022 ▸) have dihedral angles between the aromatic rings of the biphenyl carb­oxy­lic acid group ranging from 40.99 (2) to 44.58 (3)°. In three compounds, ILURAL (Hurlock *et al.*, 2021 ▸), QAKHOD (O’Malley *et al.*, 2021 ▸ and RADDIN (Doiron *et al.*, 2020 ▸), one of the dihedral angles lies between 54.71 (3) and 59.70 (6)°. In the title compound, this dihedral angle is 26.09 (4)°. The relatively small dihedral angle may be attributed to the presence of the bulky benz­yloxy group attached to the biphenyl carb­oxy­lic acid group and may also be a result of the tensile force between the two dimers. For mol­ecules containing the benz­yloxy fragment, a search resulted in thirty matches: in all of these, the torsion angle of the linking C—O—C—C unit indicates a conformation close to *anti*.

## 5. Synthesis and crystallization

Methyl 4′-(benz­yloxy)-[1,1′-biphen­yl]-3-carboxyl­ate was added in a round-bottom flask containing a solution (5%, 1.25 g of KOH in 25 mL of ethanol) of potassium hydroxide in water and a small excess amount of ethyl alcohol. The whole reaction mixture was refluxed at 373 K for 6 h, cooled and poured into ice-cold hydro­chloric acid. The product 4′-(benz­yloxy)-[1,1′-biphen­yl]-3-carb­oxy­lic acid separated out as a solid, which was filtered and then washed with water to remove excess hydro­chloric acid. Finally, single crystals suitable for X-ray diffraction studies were grown in pure ethanol at room temperature. For the detailed synthesis procedure, see Radhika *et al.* (2011 ▸). ^1^H NMR: (CDCl_3_, δ): 12 (*s*, 1H, –COOH), 8.74-8.24 (*m*, 2H, Ar-H), 7.85–7.78 (*m*, 4H, Ar-H), 7.48–7.02 (*m*, 7H, Ar-H), 5.0 (*s*, 2H, –OCH_2_–) ppm.

## 6. Hirshfeld surface analysis

**Figure 4:**
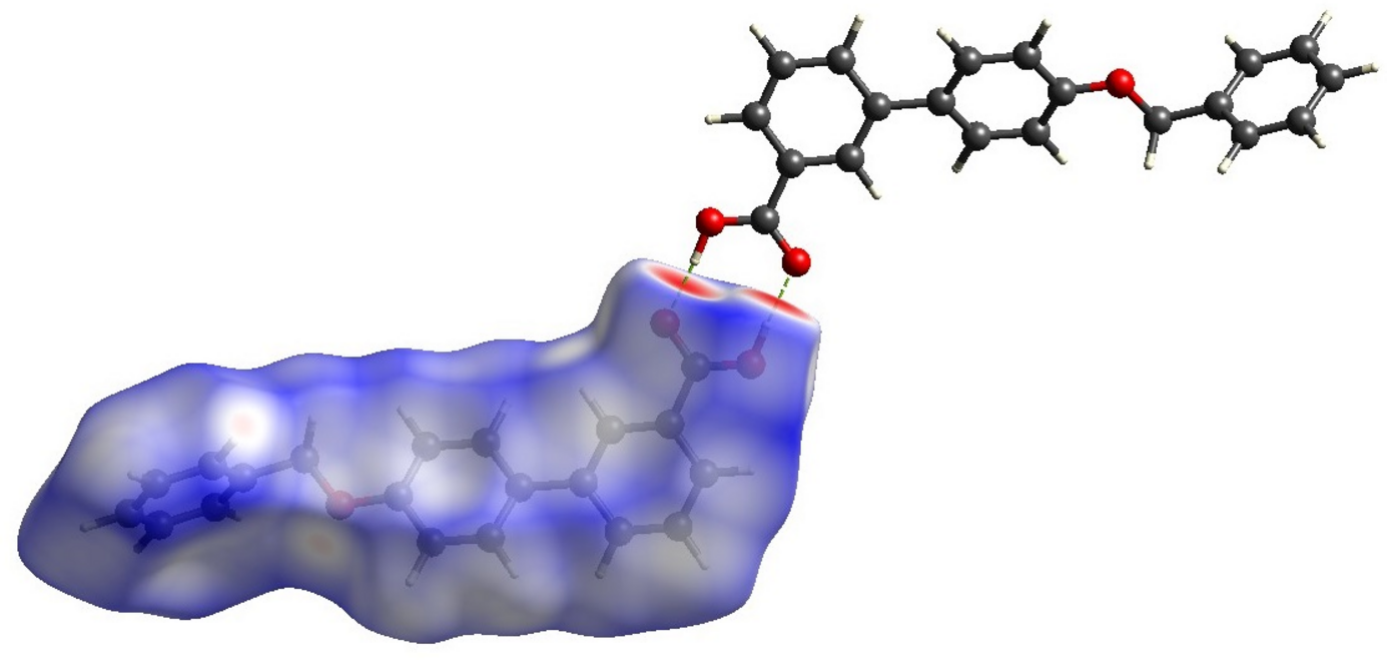
The Hirshfeld surface of the title compound with the dashed lines indicating the O—H⋯O hydrogen bonds that form inversion dimers.

**Figure 5:**
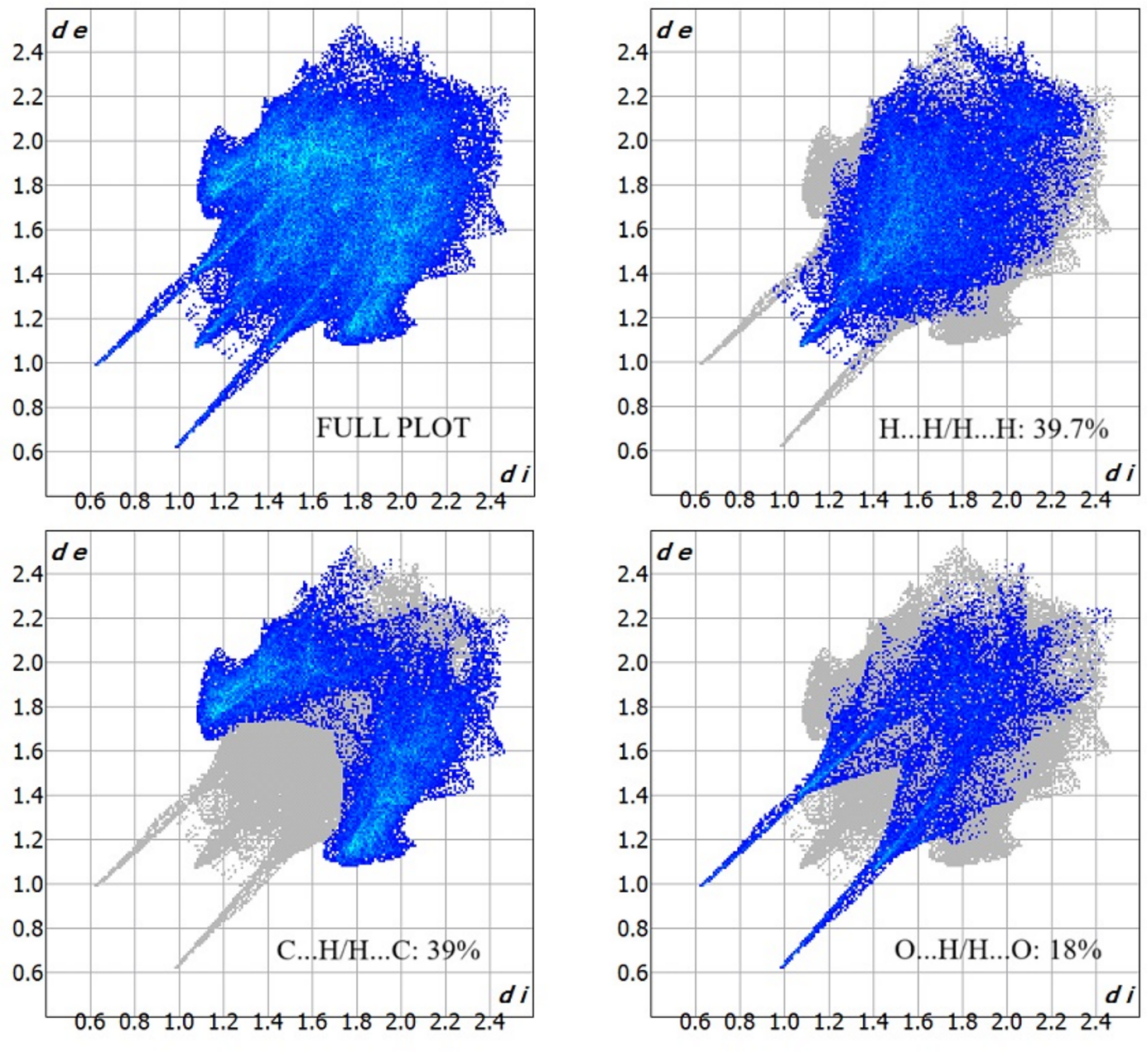
The two-dimensional fingerprint plots of the title mol­ecule showing all inter­actions and those delineated into H⋯H, C⋯H/H⋯C and H⋯O/O⋯H.

**Figure 6:**
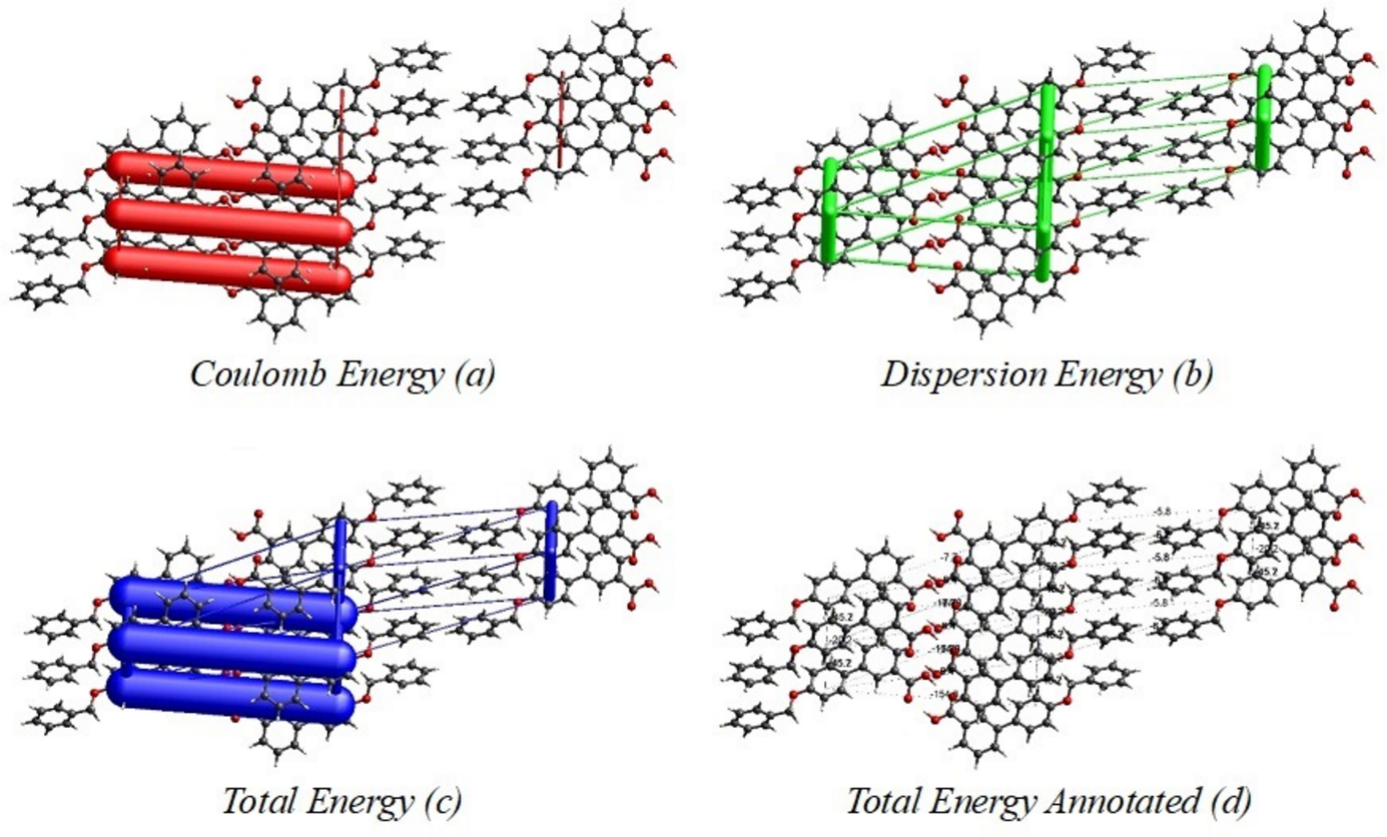
The energy frameworks for inter­action energies in the title compound, (*a*) Coulombic energy, (*b*) dispersion energy, (*c*) total energy and (*d*) total energy annotated.

Hirshfeld surface analysis (Hirshfeld, 1977 ▸; Spackman & Jayatilaka, 2009 ▸) was performed to visualize and qu­antify the inter­molecular inter­actions in the title mol­ecule using *CrystalExplorer* (Spackman *et al.*, 2021 ▸). The Hirshfeld surface mapped over *d*_norm_ is shown in Fig. 4 ▸ with colors representing inter­molecular inter­actions on the surface. The red regions are attributed to the O2—H2⋯O3 inter­action. The two-dimensional fingerprint plots indicate that the major contributions to the crystal packing are from H⋯H (39.7%), C⋯H/H⋯C (39%) and O⋯H/H⋯O (18%) as shown in Fig. 5 ▸. The net inter­action energies were calculated as *E*_ele_ = 145.6 kJ mol^−1^, *E*_pol_ = 47.3 kJ mol^−1^, *E*_dis_ = 201.0 kJ mol^−1^, *E*_rep_ = 83.6 kJ mol^−1^ and total inter­action energy *E*_tot_ = 308.0 kJ mol^−1^. The topology of the energy frameworks for the Coulombic, dispersion and total energies are shown in Fig. 6 ▸. Higher dispersion energy can affect the reactivity of the mol­ecules, particularly in biological processes such as docking the ligand with a protein. The dispersion energy influences the binding affinity of the ligand by providing an additional attractive force.

## 7. DFT Studies

**Figure 7:**
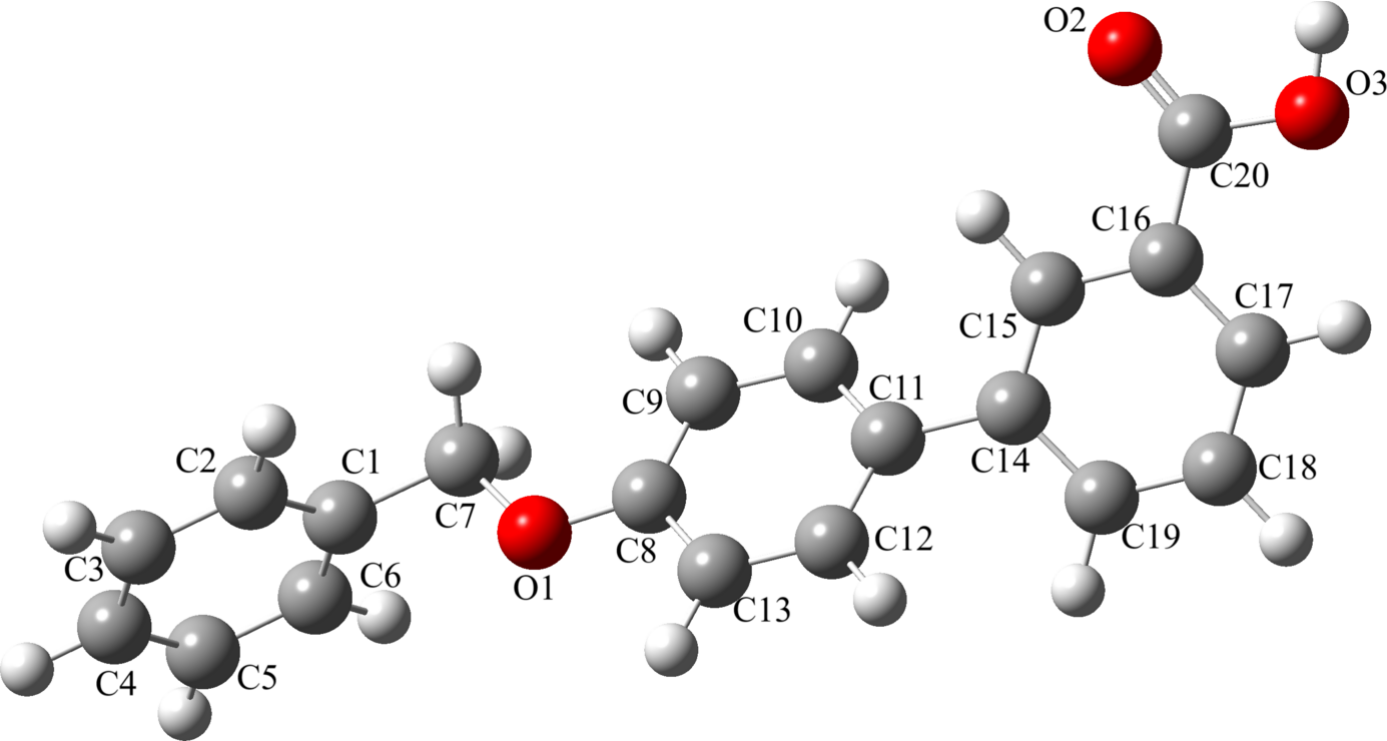
The optimized structure of the title compound generated using *Gaussian 09* at the B3LYP/6–311++G(d,p) level.

**Figure 8:**
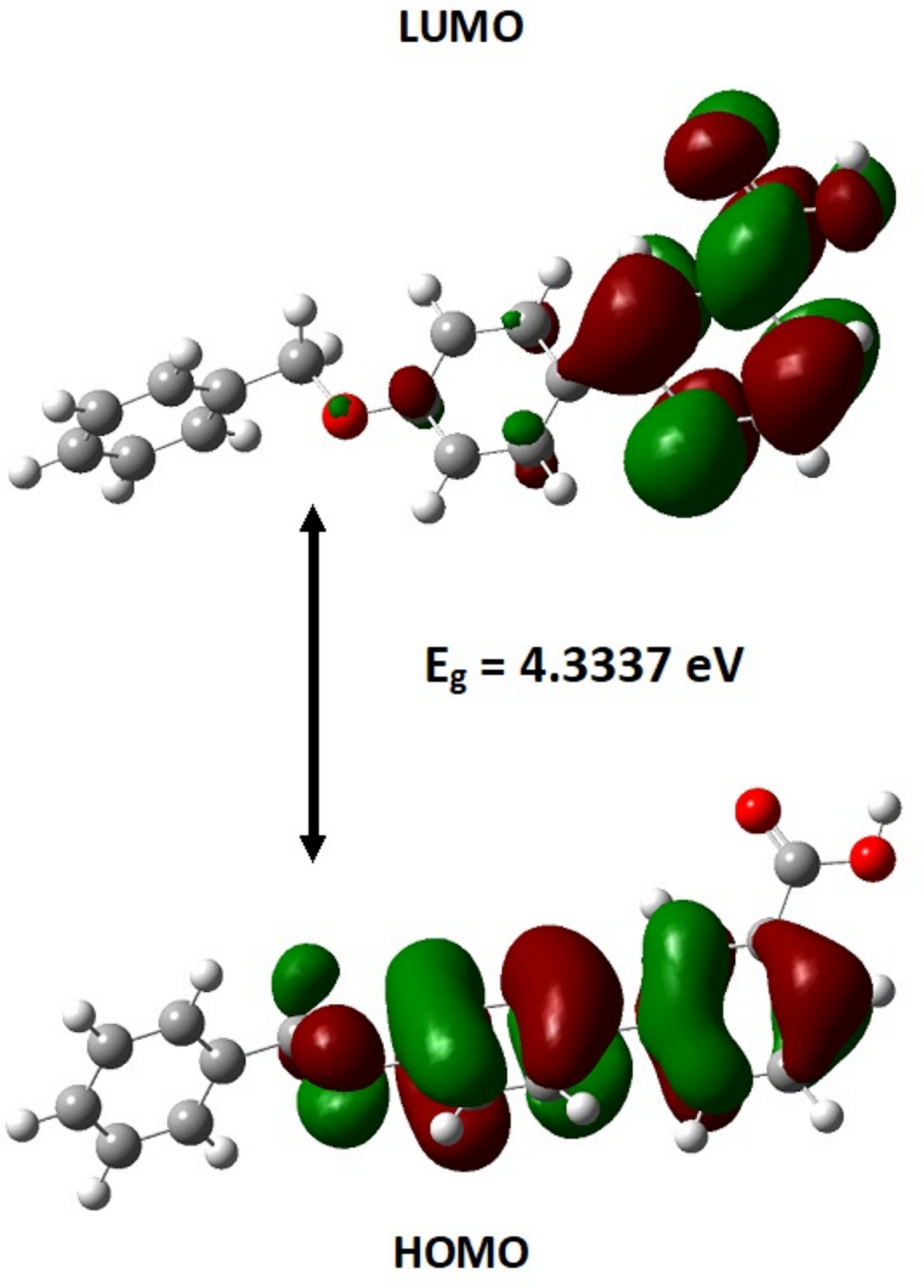
The HOMO and LUMO mol­ecular orbitals of the title compound.

The HOMO–LUMO levels are valuable for understanding the mol­ecule’s inter­actions in chemical reactions, electronic transitions, and stability. The mol­ecule was constructed using *Gaussview 06* and optimized with the B3LYP/6-311++G(d,p) model in *Gaussian 09* (Frisch *et al.*, 2009 ▸). The optimized structure is illustrated in Fig. 7 ▸. The optimized bond lengths, angles and torsion angles were compared with those obtained from SCXRD data (Table 2 ▸) and are found to be in good agreement with each other. The tensile force between the two dimers is not taken into the account in the quantum calculations, so there is a common donor–hydrogen distance around the carb­oxy­lic group in the DFT calculations. Fig. 8 ▸ shows the HOMO and LUMO and their energy gap in the title compound. In the HOMO, electron density is mainly concentrated on the biphenyl rings, with a smaller presence on the oxygen atom in the benz­yloxy group. In the LUMO, the electron density is primarily located on the benzoic acid portion of the biphenyl group. The HOMO and LUMO energies are −6.0814 eV and −1.7466 eV, respectively, resulting in an energy gap (Δ*E*) of 4.3347 eV. Reactivity descriptors including ionization energy (I), electron affinity (A), electronegativity (χ), chemical hardness (η), chemical potential (μ), electrophilicity index (ω), and chemical softness (S) are presented in Table 3 ▸. The electrophilicity index value of 3.534 eV indicates that the mol­ecule exhibits strong electrophilicity.

## 8. Mol­ecular electrostatic potential

**Figure 9:**
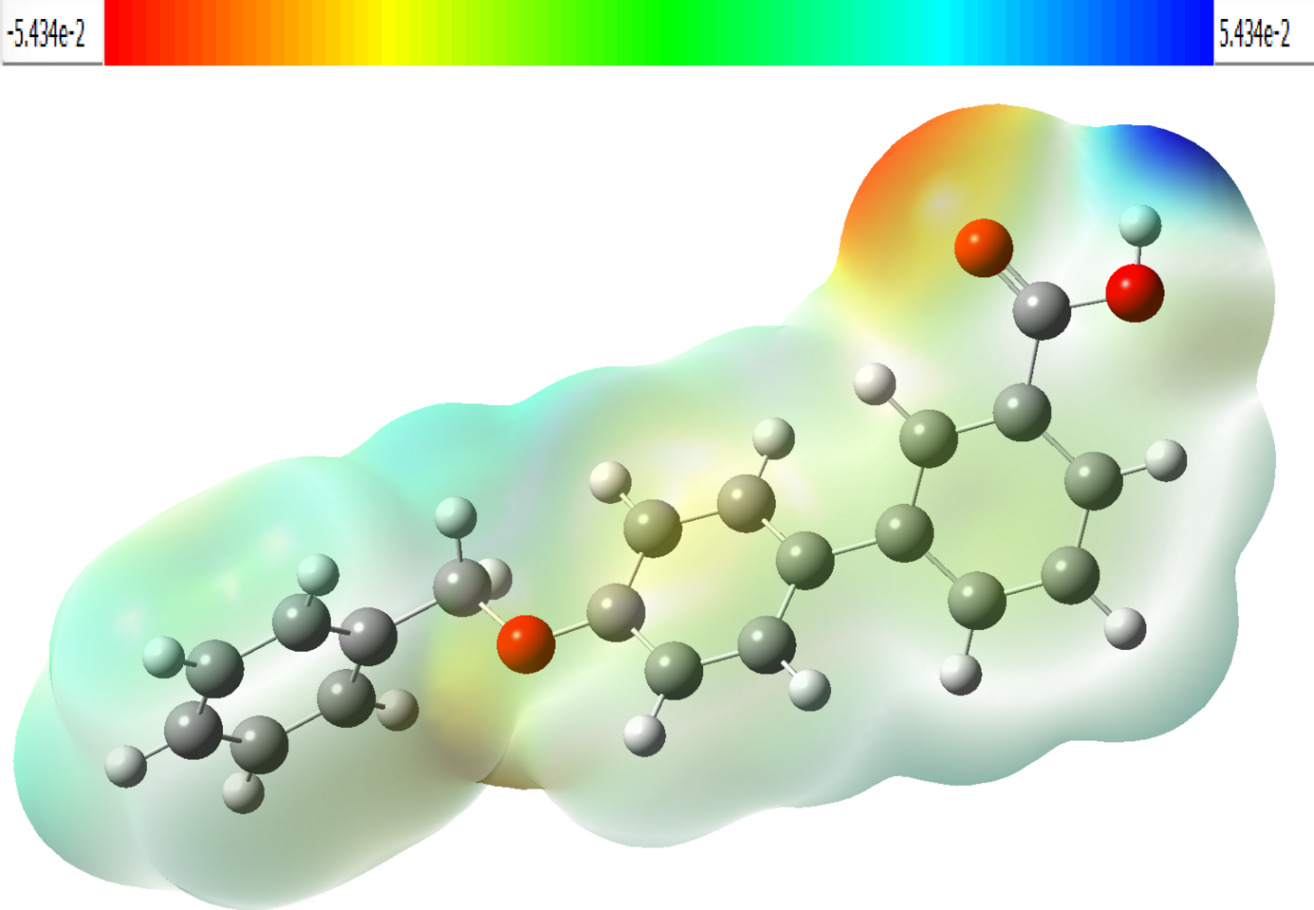
The mol­ecular electrostatic potential surface of the title compound.

The mol­ecular electrostatic potential surface (MEPS) helps to visualize the electrostatic environment around a mol­ecule and is illustrated for the title compound in Fig. 9 ▸. The electron-rich part with a partial negative charge is shown by the combination of red and pale-yellow regions on the MEPS over the oxygen atom of the carb­oxy­lic acid moiety and is an active site for electrophilic attack, which is essential for biological recognition and hydrogen-bonding interactions. The bright-blue region on the MEPS over the hydrogen atom of the carb­oxy­lic acid moiety is an active site for possible nucleophilic attack (Friesner *et al.*, 2006 ▸).

## 9. Mol­ecular docking studies

**Figure 10:**
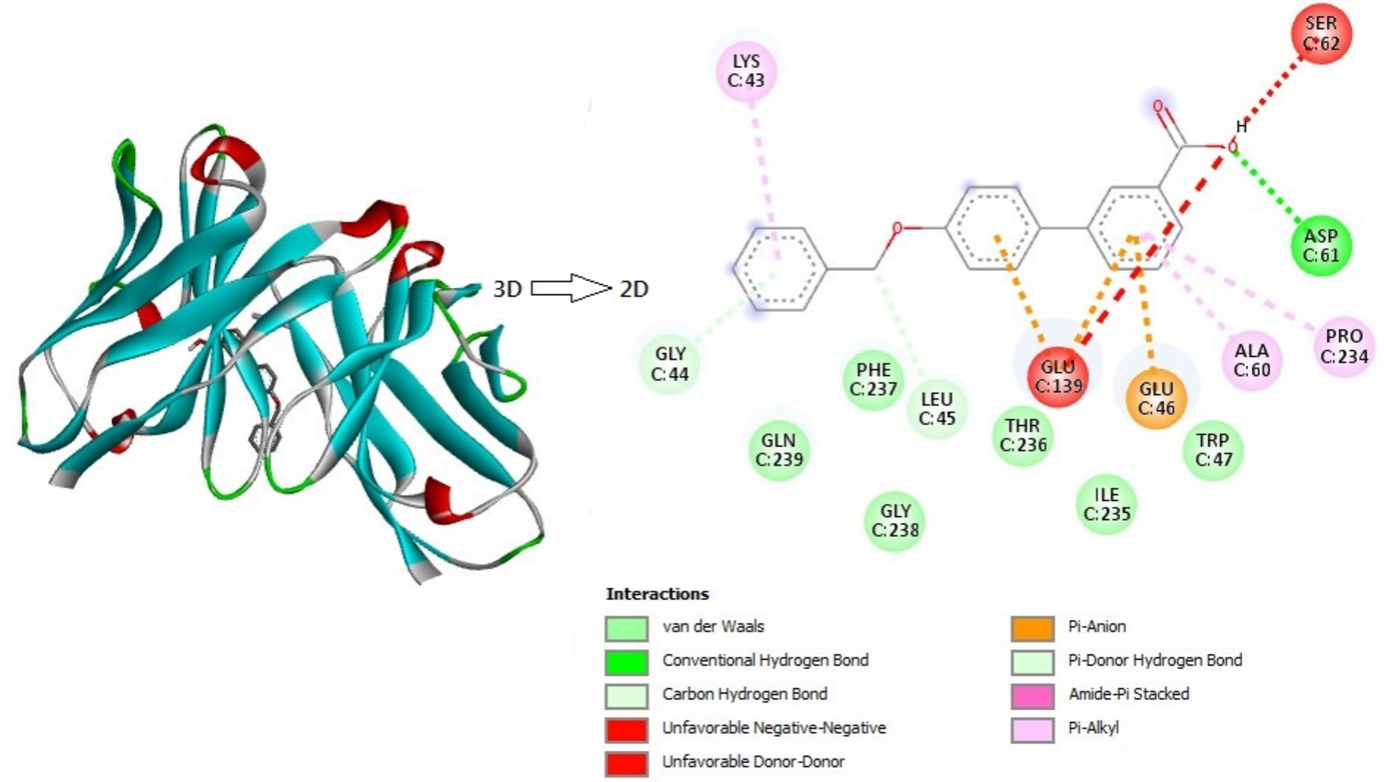
The three-dimensional and two-dimensional views of various inter­actions between the title mol­ecule (ligand) and the receptor protein SARS-Covid-2 (PDB ID:8BEC).

The docking of a receptor protein, specifically the Omicron variant (PDB ID:8BEC, SARS-COV2-VARIANT), with the synthesized ligand shows a very good binding affinity of −7.6 kcal mol^−1^. *AutoDock Vina* (Morris *et al.*, 2009 ▸) was used for theoretical calculations and the inter­action was generated by *Discovery Studio Visualizer* (Biovia, 2017 ▸). A 2D view of the docking inter­actions shows one conventional bond with ACP C:61 and two π-donor hydrogen bonds with GLY C:44 and LEU C:45. The higher dispersion energy influences the ligand to have conformational stability with the protein. The idea of docking of the protein mol­ecules with the centroids of the ligands can be used in structure-based drug design. Modifications in the synthesized ligands by varying functional groups and atoms can easily achieve a very good binding affinity with the target protein. In the title ligand we can see three centroids, of which *Cg*1 and *Cg*2 (the centroids of the C1–C6 and C8–C12 rings) play significant role in the inter­molecular inter­actions. Meanwhile these act as anchor points for the ligand, the inter­action with these centroids and GLU C:139, GLU C:46 and ALU C:60 PRO C:234 amino acids forming π–anion and π–donor hydrogen bonds, respectively. In addition to these inter­actions, a few van der Waals inter­actions can be seen around the ligand and unfavorable inter­actions are observed at the –OH group; these are shown in Fig. 10 ▸.

## 10. Refinement

Crystal data, data collection and structure refinement details are summarized in Table 4 ▸. The hydrogen atom of the hydroxyl group was freely refined. All other H atoms were positioned with idealized geometry and refined using a riding model with C—H = 0.93–0.97 Å and *U*_iso_(H) = 1.2*U*_eq_(C) or 1.5*U*_eq_(methyl C).

## Supplementary Material

Crystal structure: contains datablock(s) I. DOI: 10.1107/S2056989025001021/nx2019sup1.cif

Supporting information file. DOI: 10.1107/S2056989025001021/nx2019Isup2.cml

CCDC reference: 2421495

Additional supporting information:  crystallographic information; 3D view; checkCIF report

## Figures and Tables

**Table 1:** Hydrogen-bond geometry (Å, °) *Cg*1 and *Cg*2 are the centroids of the C1–C6 and C8–C12 rings, respectively,

*D*—H⋯*A*	*D*—H	H⋯*A*	*D*⋯*A*	*D*—H⋯*A*
C15—H15⋯O2	0.93	2.48	2.791 (3)	100
C17—H17⋯O3	0.93	2.48	2.767 (4)	98
O3—H3*A*⋯O2^i^	1.20 (5)	1.42 (5)	2.617 (3)	175 (4)
C3—H3⋯*Cg*1^ii^	0.93	2.88	3.711 (4)	149
C6—H6⋯*Cg*1^iii^	0.93	2.77	3.588 (4)	147
C9—H9⋯*Cg*2^iv^	0.93	2.86	3.667 (3)	146
C12—H12⋯*Cg*2^v^	0.93	2.81	3.629 (3)	147

Symmetry codes: (i) 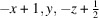; (ii) 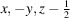; (iii) 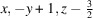; (iv) 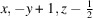; (v) 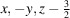.

**Table 2:** Selected bond lengths, angles and torsion angles (Å, °)

Parameter	SCXRD	DFT
O1—C8	1.366 (3)	1.36392
O1—C7	1.437 (3)	1.43631
O2—C20	1.241 (3)	1.20944
O3—C20	1.270 (3)	1.35882
C8—O1—C7	117.75 (19)	118.64625
O1—C8—C13	115.7 (2)	115.79634
O1—C8—C9	125.3 (2)	124.91696
C13—C8—C9	119.0 (2)	119.28680
C7—O1—C8—C13	179.0 (2)	179.2209
C7—O1—C8—C9	−0.5 (4)	−0.79351
C8—O1—C7—C1	−175.9 (2)	−178.9820
O1—C8—C13—C12	−178.5 (2)	−179.9223

**Table 3:** The energy values (eV) of global reactivity descriptors for the title compound

*E*_HOMO_	−6.0801
*E*_LUMO_	−1.7464
Energy gap (eV)	4.3337
Ionization Energy (*I*)	6.0801
Electron affinity (*A*)	1.7464
Electronegativity (χ)	3.91325
Chemical hardness (η)	2.16685
Chemical softness (*S*)	0.231 eV^−1^
Chemical potential (μ)	−3.91325
Electrophilicity index (ω)	3.534

**Table 4:** Experimental details

Crystal data	
Chemical formula	C_20_H_16_O_3_
*M*_r_	304.33
Crystal system, space group	Monoclinic, *P*2/*c*
Temperature (K)	299
*a*, *b*, *c* (Å)	31.9237 (13), 7.0199 (3), 6.9184 (3)
β (°)	91.864 (1)
*V* (Å^3^)	1549.60 (11)
*Z*	4
Radiation type	Mo *K*α
μ (mm^−1^)	0.09
Crystal size (mm)	0.31 × 0.27 × 0.18

Data collection	
Diffractometer	Bruker *SMART* APEXII CCD
Absorption correction	Multi-scan (*SADABS*; Krause *et al.*, 2015 ▸)
*T*_min_, *T*_max_	0.972, 0.983
No. of measured, independent and observed [*I* > 2σ(*I*)] reflections	35610, 2760, 2207
*R*_int_	0.072
(sin θ/λ)_max_ (Å^−1^)	0.597

Refinement	
*R*[*F*^2^ > 2σ(*F*^2^)], *wR*(*F*^2^), *S*	0.069, 0.162, 1.13
No. of reflections	2760
No. of parameters	212
H-atom treatment	H atoms treated by a mixture of independent and constrained refinement
Δρ_max_, Δρ_min_ (e Å^−3^)	0.18, −0.20

Computer programs: *APEX2* and *SAINT* (Bruker, 2017 ▸), *SHELXT2018* (Sheldrick, 2015*a* ▸), *SHELXL2019/2* (Sheldrick, 2015*b* ▸) and *Mercury* (Macrae *et al.*, 2020 ▸).

## supplementary crystallographic information

### 3-[4-(Benzyloxy)phenyl]benzoic acid Crystal data

**Table:**

C_20_H_16_O_3_	*F*(000) = 640
*M**_r_* = 304.33	*D*_x_ = 1.304 Mg m^−^^3^
Monoclinic, *P*2/*c*	Mo *K*α radiation, λ = 0.71073 Å
Hall symbol: -P 2yc	Cell parameters from 2207 reflections
*a* = 31.9237 (13) Å	θ = 2.0–25.0°
*b* = 7.0199 (3) Å	µ = 0.09 mm^−^^1^
*c* = 6.9184 (3) Å	*T* = 299 K
β = 91.864 (1)°	Prism, colourless
*V* = 1549.60 (11) Å^3^	0.31 × 0.27 × 0.18 mm
*Z* = 4	

### 3-[4-(Benzyloxy)phenyl]benzoic acid Data collection

**Table:**

Bruker SMART APEXII CCD diffractometer	2760 independent reflections
Radiation source: fine-focus sealed tube	2207 reflections with *I* > 2σ(*I*)
Graphite monochromator	*R*_int_ = 0.072
Detector resolution: 1.09 pixels mm^-1^	θ_max_ = 25.1°, θ_min_ = 2.6°
φ and Ω scans	*h* = −38→38
Absorption correction: multi-scan (SADABS; Krause *et al.*, 2015)	*k* = −8→8
*T*_min_ = 0.972, *T*_max_ = 0.983	*l* = −8→8
35610 measured reflections	

### 3-[4-(Benzyloxy)phenyl]benzoic acid Refinement

**Table:**

Refinement on *F*^2^	Primary atom site location: structure-invariant direct methods
Least-squares matrix: full	Secondary atom site location: difference Fourier map
*R*[*F*^2^ > 2σ(*F*^2^)] = 0.069	Hydrogen site location: mixed
*wR*(*F*^2^) = 0.162	H atoms treated by a mixture of independent and constrained refinement
*S* = 1.13	*w* = 1/[σ^2^(*F*_o_^2^) + (0.0584*P*)^2^ + 1.0677*P*] where *P* = (*F*_o_^2^ + 2*F*_c_^2^)/3
2760 reflections	(Δ/σ)_max_ < 0.001
212 parameters	Δρ_max_ = 0.18 e Å^−^^3^
0 restraints	Δρ_min_ = −0.20 e Å^−^^3^
0.12 constraints	

### 3-[4-(Benzyloxy)phenyl]benzoic acid Special details

**Table:**

Geometry. All esds (except the esd in the dihedral angle between two l.s. planes) are estimated using the full covariance matrix. The cell esds are taken into account individually in the estimation of esds in distances, angles and torsion angles; correlations between esds in cell parameters are only used when they are defined by crystal symmetry. An approximate (isotropic) treatment of cell esds is used for estimating esds involving l.s. planes.

### 3-[4-(Benzyloxy)phenyl]benzoic acid Fractional atomic coordinates and isotropic or equivalent isotropic displacement parameters (Å^2^)

**Table:**

	*x*	*y*	*z*	*U*_iso_*/*U*_eq_	
O1	0.82388 (5)	0.2450 (3)	0.2817 (2)	0.0451 (5)	
O2	0.55270 (6)	0.2739 (4)	0.2404 (3)	0.0803 (8)	
O3	0.51314 (6)	0.2653 (5)	0.4950 (3)	0.0951 (10)	
C8	0.78393 (7)	0.2524 (3)	0.3464 (3)	0.0348 (6)	
C12	0.73839 (7)	0.1552 (4)	0.5975 (3)	0.0383 (6)	
H12	0.734753	0.091923	0.713867	0.046*	
C13	0.77746 (8)	0.1606 (4)	0.5216 (4)	0.0388 (6)	
H13	0.799867	0.102365	0.587397	0.047*	
C14	0.66164 (7)	0.2393 (3)	0.5886 (3)	0.0365 (6)	
C10	0.71123 (8)	0.3348 (4)	0.3318 (4)	0.0388 (6)	
H10	0.688949	0.394984	0.266940	0.047*	
C11	0.70391 (7)	0.2422 (3)	0.5053 (3)	0.0348 (6)	
C9	0.75043 (7)	0.3408 (4)	0.2519 (4)	0.0391 (6)	
H9	0.754192	0.403897	0.135543	0.047*	
C15	0.62556 (8)	0.2555 (4)	0.4737 (4)	0.0431 (6)	
H15	0.628038	0.268632	0.340789	0.052*	
C16	0.58606 (8)	0.2530 (4)	0.5484 (4)	0.0451 (7)	
C1	0.87677 (8)	0.2945 (4)	0.0553 (4)	0.0417 (6)	
C6	0.90842 (9)	0.4146 (4)	0.1193 (4)	0.0522 (7)	
H6	0.901833	0.522717	0.189822	0.063*	
C19	0.65636 (9)	0.2203 (4)	0.7877 (4)	0.0468 (7)	
H19	0.679840	0.208984	0.870101	0.056*	
C7	0.83194 (8)	0.3350 (4)	0.1001 (4)	0.0487 (7)	
H7A	0.827368	0.471270	0.108922	0.058*	
H7B	0.813364	0.284253	−0.001031	0.058*	
C17	0.58190 (9)	0.2345 (4)	0.7471 (4)	0.0540 (8)	
H17	0.555481	0.233411	0.799881	0.065*	
C20	0.54871 (9)	0.2661 (5)	0.4180 (4)	0.0566 (8)	
C2	0.88725 (9)	0.1367 (4)	−0.0504 (4)	0.0528 (7)	
H2	0.866309	0.054912	−0.096613	0.063*	
C18	0.61711 (9)	0.2180 (5)	0.8637 (4)	0.0566 (8)	
H18	0.614462	0.205111	0.996566	0.068*	
C5	0.94942 (9)	0.3774 (5)	0.0806 (4)	0.0626 (9)	
H5	0.970380	0.459793	0.125360	0.075*	
C3	0.92875 (10)	0.0985 (5)	−0.0889 (5)	0.0626 (9)	
H3	0.935578	−0.009465	−0.159101	0.075*	
C4	0.95967 (9)	0.2195 (5)	−0.0237 (4)	0.0617 (9)	
H4	0.987522	0.194607	−0.050102	0.074*	
H3A	0.4838 (16)	0.265 (7)	0.382 (7)	0.155 (19)*	

### 3-[4-(Benzyloxy)phenyl]benzoic acid Atomic displacement parameters (Å^2^)

**Table:**

	*U*^11^	*U*^22^	*U*^33^	*U*^12^	*U*^13^	*U*^23^
O1	0.0345 (10)	0.0588 (12)	0.0423 (10)	0.0053 (8)	0.0067 (7)	0.0124 (9)
O2	0.0398 (12)	0.157 (3)	0.0447 (13)	−0.0007 (13)	0.0056 (9)	0.0059 (14)
O3	0.0307 (11)	0.199 (3)	0.0562 (14)	0.0012 (15)	0.0094 (10)	−0.0046 (17)
C8	0.0322 (12)	0.0346 (13)	0.0379 (13)	−0.0004 (10)	0.0043 (10)	−0.0009 (11)
C12	0.0399 (14)	0.0415 (14)	0.0336 (13)	0.0013 (11)	0.0038 (10)	0.0057 (11)
C13	0.0373 (14)	0.0416 (14)	0.0372 (14)	0.0057 (11)	−0.0036 (11)	0.0057 (11)
C14	0.0389 (14)	0.0332 (13)	0.0378 (13)	−0.0023 (11)	0.0049 (11)	−0.0026 (11)
C10	0.0352 (13)	0.0431 (15)	0.0381 (14)	0.0049 (11)	0.0009 (10)	0.0062 (12)
C11	0.0365 (13)	0.0327 (13)	0.0353 (13)	−0.0019 (11)	0.0025 (10)	−0.0020 (11)
C9	0.0384 (14)	0.0410 (14)	0.0383 (14)	0.0035 (11)	0.0045 (11)	0.0079 (12)
C15	0.0371 (14)	0.0558 (17)	0.0366 (14)	−0.0009 (12)	0.0059 (11)	−0.0024 (13)
C16	0.0390 (14)	0.0552 (17)	0.0414 (15)	−0.0005 (12)	0.0077 (11)	−0.0035 (13)
C1	0.0416 (14)	0.0467 (15)	0.0371 (14)	−0.0021 (12)	0.0068 (11)	0.0070 (12)
C6	0.0550 (17)	0.0605 (18)	0.0415 (15)	−0.0071 (14)	0.0090 (13)	−0.0110 (14)
C19	0.0480 (16)	0.0544 (17)	0.0381 (15)	−0.0021 (13)	0.0032 (12)	−0.0010 (13)
C7	0.0443 (15)	0.0581 (17)	0.0442 (16)	0.0034 (13)	0.0101 (12)	0.0178 (14)
C17	0.0401 (15)	0.074 (2)	0.0490 (17)	−0.0001 (14)	0.0167 (13)	−0.0016 (15)
C20	0.0344 (15)	0.085 (2)	0.0509 (18)	0.0000 (15)	0.0084 (12)	−0.0004 (16)
C2	0.0525 (17)	0.0566 (18)	0.0496 (17)	−0.0112 (14)	0.0054 (13)	−0.0063 (14)
C18	0.0515 (18)	0.081 (2)	0.0379 (15)	−0.0022 (15)	0.0094 (13)	−0.0013 (15)
C5	0.0447 (17)	0.087 (2)	0.0561 (19)	−0.0173 (16)	0.0053 (14)	−0.0050 (18)
C3	0.066 (2)	0.062 (2)	0.060 (2)	0.0091 (16)	0.0209 (16)	−0.0060 (16)
C4	0.0433 (17)	0.090 (3)	0.0530 (18)	0.0062 (17)	0.0166 (14)	0.0098 (18)

### 3-[4-(Benzyloxy)phenyl]benzoic acid Geometric parameters (Å, º)

**Table:**

O1—C8	1.366 (3)	C16—C20	1.474 (4)
O1—C7	1.437 (3)	C1—C2	1.375 (4)
O2—C20	1.241 (3)	C1—C6	1.378 (4)
O3—C20	1.270 (3)	C1—C7	1.501 (4)
O3—H3A	1.20 (5)	C6—C5	1.370 (4)
C8—C9	1.382 (3)	C6—H6	0.9300
C8—C13	1.394 (3)	C19—C18	1.375 (4)
C12—C13	1.369 (3)	C19—H19	0.9300
C12—C11	1.395 (3)	C7—H7A	0.9700
C12—H12	0.9300	C7—H7B	0.9700
C13—H13	0.9300	C17—C18	1.367 (4)
C14—C15	1.383 (4)	C17—H17	0.9300
C14—C19	1.400 (3)	C2—C3	1.386 (4)
C14—C11	1.485 (3)	C2—H2	0.9300
C10—C9	1.385 (3)	C18—H18	0.9300
C10—C11	1.391 (3)	C5—C4	1.368 (5)
C10—H10	0.9300	C5—H5	0.9300
C9—H9	0.9300	C3—C4	1.368 (4)
C15—C16	1.378 (3)	C3—H3	0.9300
C15—H15	0.9300	C4—H4	0.9300
C16—C17	1.391 (4)		
			
C8—O1—C7	117.75 (19)	C18—C19—C14	121.2 (3)
C20—O3—H3A	115 (2)	C18—C19—H19	119.4
O1—C8—C9	125.3 (2)	C14—C19—H19	119.4
O1—C8—C13	115.7 (2)	O1—C7—C1	107.2 (2)
C9—C8—C13	119.0 (2)	O1—C7—H7A	110.3
C13—C12—C11	121.8 (2)	C1—C7—H7A	110.3
C13—C12—H12	119.1	O1—C7—H7B	110.3
C11—C12—H12	119.1	C1—C7—H7B	110.3
C12—C13—C8	120.6 (2)	H7A—C7—H7B	108.5
C12—C13—H13	119.7	C18—C17—C16	119.2 (2)
C8—C13—H13	119.7	C18—C17—H17	120.4
C15—C14—C19	116.7 (2)	C16—C17—H17	120.4
C15—C14—C11	121.8 (2)	O2—C20—O3	122.5 (3)
C19—C14—C11	121.6 (2)	O2—C20—O3	122.5 (3)
C9—C10—C11	122.5 (2)	O2—C20—O3	122.5 (3)
C9—C10—H10	118.7	O2—C20—O3	122.5 (3)
C11—C10—H10	118.7	O2—C20—C16	120.1 (2)
C10—C11—C12	116.6 (2)	O2—C20—C16	120.1 (2)
C10—C11—C14	121.4 (2)	O3—C20—C16	117.4 (3)
C12—C11—C14	122.0 (2)	O3—C20—C16	117.4 (3)
C8—C9—C10	119.5 (2)	C1—C2—C3	120.6 (3)
C8—C9—H9	120.2	C1—C2—H2	119.7
C10—C9—H9	120.2	C3—C2—H2	119.7
C16—C15—C14	122.6 (2)	C17—C18—C19	121.0 (3)
C16—C15—H15	118.7	C17—C18—H18	119.5
C14—C15—H15	118.7	C19—C18—H18	119.5
C15—C16—C17	119.3 (3)	C4—C5—C6	120.3 (3)
C15—C16—C20	120.1 (2)	C4—C5—H5	119.9
C17—C16—C20	120.6 (2)	C6—C5—H5	119.9
C2—C1—C6	118.4 (3)	C4—C3—C2	120.0 (3)
C2—C1—C7	120.8 (3)	C4—C3—H3	120.0
C6—C1—C7	120.8 (3)	C2—C3—H3	120.0
C5—C6—C1	121.1 (3)	C3—C4—C5	119.6 (3)
C5—C6—H6	119.5	C3—C4—H4	120.2
C1—C6—H6	119.5	C5—C4—H4	120.2
			
C7—O1—C8—C9	−0.5 (4)	C8—O1—C7—C1	−175.9 (2)
C7—O1—C8—C13	179.0 (2)	C2—C1—C7—O1	90.9 (3)
C11—C12—C13—C8	−0.7 (4)	C6—C1—C7—O1	−89.1 (3)
O1—C8—C13—C12	−178.5 (2)	C15—C16—C17—C18	−0.4 (5)
C9—C8—C13—C12	1.0 (4)	C20—C16—C17—C18	178.4 (3)
C9—C10—C11—C12	0.4 (4)	C15—C16—C20—O2	2.1 (5)
C9—C10—C11—C14	179.5 (2)	C17—C16—C20—O2	−176.8 (3)
C13—C12—C11—C14	−179.1 (2)	C15—C16—C20—O2	2.1 (5)
C15—C14—C11—C10	26.6 (4)	C17—C16—C20—O2	−176.8 (3)
C19—C14—C11—C10	−153.3 (3)	C15—C16—C20—O3	−179.2 (3)
C15—C14—C11—C12	−154.4 (3)	C17—C16—C20—O3	1.9 (5)
C19—C14—C11—C12	25.7 (4)	C15—C16—C20—O3	−179.2 (3)
O1—C8—C9—C10	178.8 (2)	C17—C16—C20—O3	1.9 (5)
C13—C8—C9—C10	−0.6 (4)	C6—C1—C2—C3	1.0 (4)
C11—C10—C9—C8	−0.1 (4)	C7—C1—C2—C3	−179.1 (3)
C19—C14—C15—C16	−0.1 (4)	C16—C17—C18—C19	0.3 (5)
C11—C14—C15—C16	180.0 (2)	C14—C19—C18—C17	−0.1 (5)
C14—C15—C16—C17	0.4 (4)	C1—C6—C5—C4	0.3 (5)
C14—C15—C16—C20	−178.5 (3)	C1—C2—C3—C4	−0.9 (5)
C2—C1—C6—C5	−0.7 (4)	C2—C3—C4—C5	0.4 (5)
C7—C1—C6—C5	179.3 (3)	C6—C5—C4—C3	−0.1 (5)
C11—C14—C19—C18	179.9 (3)		

### 3-[4-(Benzyloxy)phenyl]benzoic acid Hydrogen-bond geometry (Å, º)

*Cg*1 and *Cg*2 are the centroids of the C1–C6 and C8–C12 rings, respectively,

**Table:**

*D*—H···*A*	*D*—H	H···*A*	*D*···*A*	*D*—H···*A*
C15—H15···O2	0.93	2.48	2.791 (3)	100
C17—H17···O3	0.93	2.48	2.767 (4)	98
O3—H3*A*···O2^i^	1.20 (5)	1.42 (5)	2.617 (3)	175 (4)
C3—H3···*Cg*1^ii^	0.93	2.88	3.711 (4)	149
C6—H6···*Cg*1^iii^	0.93	2.77	3.588 (4)	147
C9—H9···*Cg*2^iv^	0.93	2.86	3.667 (3)	146
C12—H12···*Cg*2^v^	0.93	2.81	3.629 (3)	147

Symmetry codes: (i) −*x*+1, *y*, −*z*+1/2; (ii) *x*, −*y*, *z*−1/2; (iii) *x*, −*y*+1, *z*−3/2; (iv) *x*, −*y*+1, *z*−1/2; (v) *x*, −*y*, *z*−3/2.
